# Growth delay of human bladder cancer cells by Prostate Stem Cell Antigen downregulation is associated with activation of immune signaling pathways

**DOI:** 10.1186/1471-2407-10-129

**Published:** 2010-04-07

**Authors:** Emanuele Marra, Paolo Uva, Valentina Viti, Valeria Simonelli, Eugenia Dogliotti, Emanuele De Rinaldis, Armin Lahm, Nicola La Monica, Alfredo Nicosia, Gennaro Ciliberto, Fabio Palombo

**Affiliations:** 1 Istituto di Ricerche Biologia Molecolare P. Angeletti (IRBM), Via Pontina Km 30,600 00040 Pomezia (Rome) Italy; 2 Okairos, Via dei Castelli Romani 22, 00040 Pomezia (Rome) Italy; 3 Istituto Superiore di Sanità (ISS), Viale Regina Elena 299, 00161 (Rome) Italy; 4 Breakthrough Breast Cancer Research Unit, King's College London, UK

## Abstract

**Background:**

Prostate stem cell antigen (PSCA) is a glycosylphosphatidylinositol (GPI) anchored protein expressed not only in prostate but also in pancreas and bladder cancer as shown by immunohistochemistry and mRNA analysis. It has been targeted by monoclonal antibodies in preclinical animal models and more recently in a clinical trial in prostate cancer patients. The biological role played in tumor growth is presently unknown. In this report we have characterized the contribution of PSCA expression to tumor growth.

**Methods:**

A bladder cell line was engineered to express a doxycycline (dox) regulated shRNA against PSCA. To shed light on the PSCA biological role in tumor growth, microarray analysis was carried out as a function of PSCA expression. Expression of gene set of interest was further analyzed by qPCR

**Results:**

Down regulation of the PSCA expression was associated with reduced cell proliferation *in vitro *and *in vivo*. Mice bearing subcutaneous tumors showed a reduced tumor growth upon treatment with dox, which effectively induced shRNA against PSCA as revealed by GFP expression. Pathway analysis of deregulated genes suggests a statistical significant association between PSCA downregulation and activation of genes downstream of the IFNα/β receptor.

**Conclusions:**

These experiments established for the first time a correlation between the level of PSCA expression and tumor growth and suggest a role of PSCA in counteracting the natural immune response.

## Background

PSCA has been discovered a decade ago and has been classified as a member of the Ly-6 family of GPI-anchored cell surface proteins [1]. It is expressed in most prostate cancer specimens, including high-grade prostatic intraepithelial neoplasia, primary androgen-dependent tumors, and hormone-refractory metastases. PSCA levels are positively correlated with Gleason grade, tumor stage, and biochemical recurrence. Its expression is also particularly elevated in bone metastasis. Finally, PSCA is strongly expressed in other malignancies, including bladder and pancreatic cancers [2-6]. Different immunotherapy approaches targeting PSCA have been tested in preclinical models including cancer vaccine, therapeutic monoclonal antibodies and antibody conjugated to toxic drugs [7-10]. More recently, a human monoclonal antibody targeting PSCA has been evaluated in a phase I clinical trial in prostate cancer patients (AACR 2006).

Little information is available regarding the biological role of PSCA. Proteins belonging to the Ly6 family are involved in cell signaling events associated with thymic lymphocyte differentiation, maturation and activation. CD59, a member of this protein family, was shown to play a role in the protection against complement mediated lysis. In addition, it was found to be expressed in tumor cells where it may play a role in evading anti cancer immune response [11]. Deletion of PSCA gene does not appear to interfere with normal development as PSCA knockout mice are viable. Additionally, crossing of the PSCA knockout mice with prostate tumor models driven by large T antigen did not increase primary tumor formation [12,13].

Here the biological role of human PSCA was evaluated using RNA interference and microarray analysis. To establish a pharmacologic control over gene expression a shRNA against PSCA was identified and expressed under the control of dox in a lentivirus system [14]. Microarray analysis was utilized to identify genes coregulated with PSCA in tumor xenografts.

## Methods

### Cell culture and generation of lentivirus vectors

The 293T and SW780 cell lines were cultured in Dulbecco's modified Eagle's medium supplemented with 10% fetal calf serum. The lentivirus system for conditional gene suppression with dox-inducible shRNAs utilized has been described previously[14]. Briefly, in the absence of dox, tTR-KRAB repressor binds to *tetO *and suppresses H1-mediated shRNA transcription, thus allowing normal expression of the cellular target gene. In the presence of 10 μg/ml of dox, tTR-KRAB cannot bind to *tetO *and hence shRNAs are produced, leading to downregulation of PSCA. The green fluorescent protein (GFP) cDNA contained in the shRNA vector provides a monitoring device, as it is switched on by dox treatment and GFPis expressed. All recombinant lentiviruses were produced by transient transfection in 293T cells. Briefly, 293T cells were cotransfected with 20 μg of pLUTHM-shPSCA3 plasmid, 15 μg of pCMV-ΔR8.91, and 5 μg of pMD2G-VSVG by calcium phosphate precipitation. After 16 h medium was changed, and recombinant lentivirus vectors were harvested 48 h later. FACS analysis was conducted as previously described [15].

### Viability assay

Cell viability was monitored using the CellTiter-Blue Viability. The assay is based on the ability of living cells to convert a redox dye (resazurin) into a fluorescent end product (resorufin); 1 × 10^3 ^SW780-shPSCA and SW780-shControl cells +/- Dox were plated in a 96 well plate in parallel with the parental cell line SW780. Cells were incubated at 37°c for 96 hrs, and fluorescence was subsequently monitored using a plate-reading fluorometer.

### Tumor models

Six-week-old female CD-1 nude mice were purchased from Charles River Laboratories and maintained in accordance to Guidelines for the Care and Use of Laboratory Animals in IRBM's animal facility. This study, was submitted and approved by the IRBM ethical committee . Mice were injected subcutaneously (sc) in the right flank with 2 × 10^6 ^SW780-shPSCA cells resuspended into 100 μL phosphate-buffered saline (PBS) and Matrigel (1:1). Mice received 5% sucrose only or 5% sucrose plus 0.2 mg/ml of dox for control and knockdown cohorts, respectively. All water bottles were changed 3 times per week. Tumors were measured with calipers and mice weighed twice per week. At the end of the dosing study, or as indicated, appropriate tumor samples were taken.

### Microarray experiments

Total RNA from cell in culture was isolated with RNAzolB and then dissolved in RNase-free water. 25 μg of total RNA was treated with DNase using the Qiagen RNase-free DNase kit and RNeasy spin columns. Then RNA was dissolved in RNase-free water to a final concentration of 0.2 μg/μl. cRNA was generated using T7 RNA polymerase on 5 μg of total RNA and labeled with Cy5 or Cy3 (Cy Dye, Amersham Pharmacia Biotech). From each sample, 5 μg of labeled-RNA were co-hybridized with 5 μg of a reference RNA (pool of two untreated SW780 cell lines). Labeled cRNAs were fragmented to an average size of 50-100 nucleotides by heating the samples to 60°C with 10 mM of zinc chloride and then adding an hybridization buffer containing 1 M NaCl, 0.5% sodium sarcosine, 50 mM MES, pH6.5, and formamide to a final concentration of 30%. The final volume was 3 ml at 40°C.

Samples were hybridized on a customized Agilent 44 k array containing ~40,000 unique probes mapping to ~21,000 human genes. Each sample was hybridized in duplicate with fluor reversal to systematically correct for dye bias. After hybridization, slides were washed and scanned using a confocal laser scanner (Agilent Technologies). The raw intensities obtained after scanning were quantified, background-corrected and lowness normalized. A weighted average ratio was computed for dye-swapped pairs of hybridizations. Tumors were collected in RNA later and processed for RNA extraction as described previously [16]. Samples were hybridized on a customized Affymetrix array containing ~38,000 probes mapping to ~21,000 human genes [17].

### Hierarchical clustering

The microarray dataset was filtered before clustering in order to select the 2,000 most variable probes. probes with an absent call in more than 12 samples out of 14 were removed the 2,000 probes with the higher interquartile range were retained for subsequent analysis. Probes were hierarchically clustered using an average linkage algorithm based on Pearson correlation coefficients.

### Identification of deregulated probes

Differences in average probe expression between the dox+ (PSCA silencing) and dox-samples were computed by 1-way ANOVA. Probes differentially expressed between the two classes were identified based on ANOVA p-value < 0.001.

### Gene set enrichment analysis

Groups of genes identified by 1-way ANOVA were compared to a collection of annotated gene sets to identify the functional classes that were significantly over-represented. The enrichment p-values were computed according to the Fisher's exact test. The gene sets were obtained from public (Gene Ontology [18], KEGG [19], Interpro [20], Panther [21], oPOSSUM [22] and commercial sources (GeneGo (GeneGo Inc., St Joseph, MI, USA), Ingenuity (Ingenuity Systems Inc, Mountain View, CA, USA), TRANSFAC [23].

### RT-PCR

Microarray findings were confirmed by real-time reverse-transcription PCR (RT-PCR) using the average value of mRNA from dox untreated SW780-shPSCA samples as calibrator. Analysis of mRNA expression of selected IFNα genes was performed using Applied Biosystems expression kit following manufacturers' instructions. Expression values were computed using the comparative CT method (ΔΔ CT) with GAPDH gene expression value serving as normaliser.

## Results

### Establishment of cell clones with inducible shRNA against PSCA

**Figure 1:**
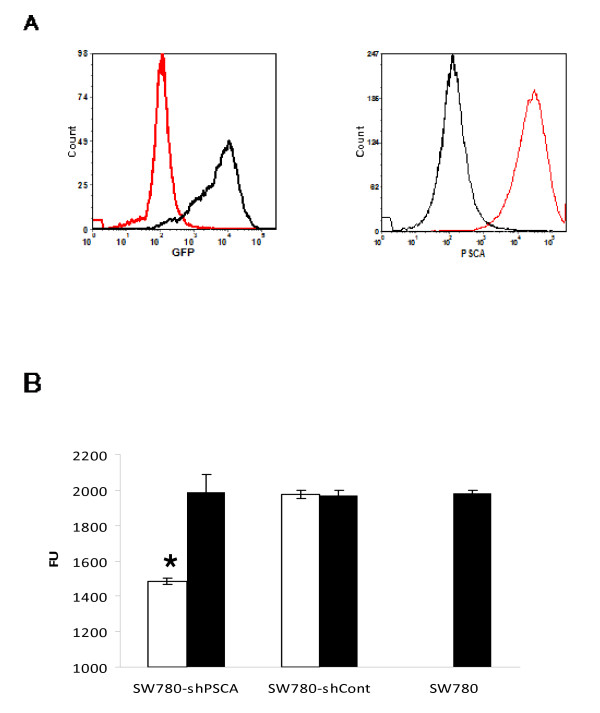
**Characterization of SW780-shPSCA cells**. **A) **FACS analysis of SW780-shPSCA cells in physiological condition (black line) or upon induction of shRNA against PSCA (red line): left panel shows GFP expression, which is a marker of shRNA expression; right panel shows PSCA expression, mean fluorescence intensity was decreased from 333 to 35 upon expression of shRNA. **B) **Viability assay in the presence of dox (empty) or without dox (filled) was carried out using the CellTiter-Blue Viability assay as described in material and methods. Error bars indicate standard error.

Published studies on PSCA have reported limited expression in cell lines engineered, for the most part, to express this protein ectopically [7,10]. Searching in a microarray cell atlas comprising 20 cell lines of different origins including prostate, pancreas and bladder revealed a low frequency of PSCA expression. The few cell lines with a detectable level of PSCA mRNA scored negative when expression was analyzed at protein level by FACS analysis (data not shown), suggesting that display of PSCA on the cell surface is uncommon. We therefore utilized a bladder cell line, SW780, which was shown to express PSCA on the cell surface [8]. The cell line was engineered to express different shRNA sequences against PSCA (Table 1) or a control shRNA against luciferase. To achieve a pharmacological controlled expression a dox inducible promoter was utilized in the context of a lentivirus system [14]. One out of six shRNA against PSCA showed a clear down regulation of target gene expression and was therefore utilized to generate SW780-shPSCA, which expresses also the tet regulator. Dox treatment resulted in 75% reduction of PSCA expression as measured by FACS analysis. Since PSCA is a membrane protein a further step was undertaken to improve regulation of gene expression by sorting out cells with low PSCA expression in the presence of dox. This process led to the isolation of cells with a larger interval of regulation mediated by dox treatment. To control that the expression of shRNA was induced upon dox treatment we took advantage of GFP gene, which is cloned upstream of the shRNA and it was previously utilized as a marker of shRNA expression [14]. FACS analysis showed a quantitative GFP expression only in dox treated SW780-shPSCA cells and a strong reduction of PSCA expression (Figure 1A). These data suggest that expression of shRNA specific for PSCA can be induced and that it leads to almost 90% downregulation of the target gene.

**Table 1:** shRNA sequences against PSCA

shRNA	Sequence	Coding Region	N
1	CATCCTAACGCAAGTCTGATT	No	497-515

2	GCAAGTCTGACCATGTATGTT	No	506-524

3	GGCAGATCAGCTCTAGTGATT	No	585-603

4	CAAGTCTGACCATGTATGTTT	No	507-525

5	GCAAGAAGAACATCACGTGTT	Yes	256-274

6	GTGACACCGACTTGTGCAATT	Yes	277-295

### Biological effects of PSCA down-regulation in SW780 cells

To characterize the impact of PSCA expression on cell growth, a viability assay was carried out. A statistical significant reduction in cell viability was observed in dox treated cells whereas no difference was observed in control transfected cells (Figure 1B).

**Figure 2:**
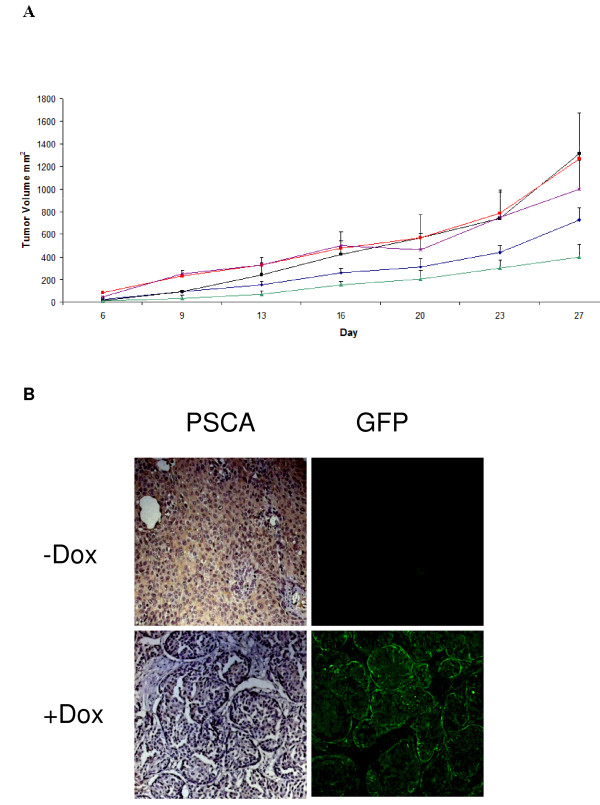
**SCA silencing inhibits SW780-shPSCA tumor growth**. **A**) Balb/C nude mice were injected s.c. with SW780-shPSCA cells or SW780-shCont cells. To further lower PSCA expression a group of mice was injected with SW780-shPSCA cells pretreated with dox *in vitro *for 1 week (green line). Groups of 6-10 mice were injected with cells and treated as follow: SW780-shPSCA bearing mice not treated with dox (red) or treated with dox (green and blue); SW780-shCont. Treated with dox (magenta) or not treated (black). **B**) At the end of the experiment SW780-shPSCA tumors were collected and subjected to IHC analysis for PSCA expression (left panel) or analyzed for GFP expression (right panel).

To verify the impact of PSCA expression on SW780-shPSCA tumor growth dox treatment was either started *in vitro *one week in advance or at the time of cell implantation. A statistical significant reduction in s.c. tumor growth (p < 0.05) was observed in the cohort of animals treated with dox with respect to controls including also SW780-shCont treated with dox (Figure 2A). Interestingly, tumors generated with cells pretreated *in vitro *with dox were smaller than tumors treated with dox only *in vivo*, although this difference was not statistically significant. In contrast control shRNA did not show any statistical reduction of tumor growth. To confirm that shRNA against PSCA was induced *in vivo *tumors were sacrificed at day 28 and expression of shRNA was evaluated by looking at GFP expression. Only dox-treated mice showed GFP expression in SW780-shPSCA tumors (Figure 2B). In line with the shRNA expression, PSCA was down-regulated as shown by IHC. All together these experiments indicate that PSCA plays a critical role in the SW780-shPSCA tumor model leading to more than 50% reduction of tumor mass with respect to dox untreated tumors.

### Transcriptional data analysis

**Figure 3:**
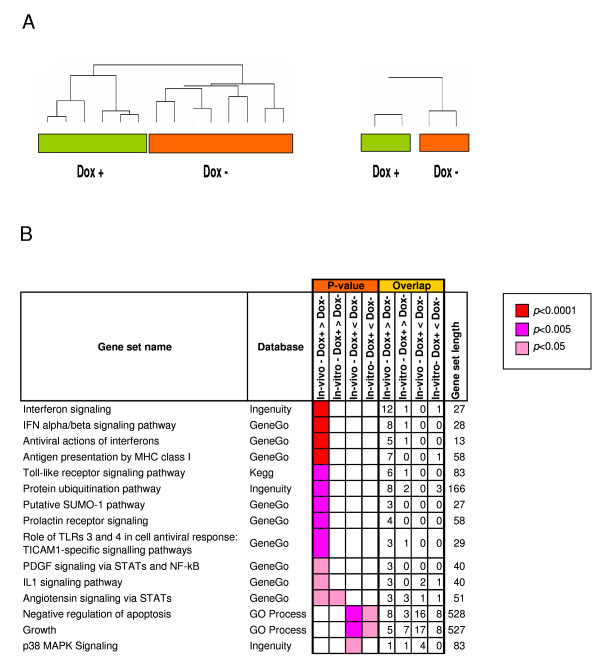
**Identification of gene classes in SW780-shPSCA tumors and cells**. To induce PSCA silencing mice or cell in culture were treated with dox (dox +) or leaved untreated (dox -). **A**) Unsuprvised hierarchical clustering of tumors and cells showed that all samples clustered according to dox treatment. Left dendogram shows results of six dox treated and eight untreated tumors collected at day 25 of tumor growth. Right dendogram shows two independent experiments conducted with cells treated for one week *in vitro *with dox and compared to untreated cells. **B) **Gene set enrichment of deregulated gene as a function of PSCA expression. Results of gene set enrichment analysis carried out in SW780-shPSCA tumors (in-vivo) or in SW780-shPSCA cells in culture (in-vitro) according to PSCA expression.

To gain some hints on the biological role played by PSCA, expression profile was carried out in tumors. Unsupervised hierarchical clustering of genome-wide expression data showed that samples clustered according to dox treatment (Figure 3A), thus demonstrating a clear effect of the PSCA expression levels on transcriptional programs. To identify the genes differentially expressed in the two experimental conditions, a 1-way ANOVA analysis was carried out. Out of the 37,586 probes represented on the array, 284 and 353 were found to be respectively up- and down-regulated in dox+ samples, as compared to dox-samples (ANOVA p-value < 0.001). Similar microarray experiments were also conducted *in vitro *showing a similar separation between the two groups, according to dox treatment. Comparison between the *in vitro *and *in vivo *data sets revealed a statistical significant overlap for the overexpressed genes (Table 2), although it may represent an underestimation due to the limited size of *in vitro *observations.

**Table 2:** Genes differentially expressed upon reduction of PSCA expression and overlap between *in vitro *and *in vivo *dataset.

Genes	*in vitro*	*in vivo*	overlap	p-value
**up regulated**	123	208	8	0.0001

**down regulated**	119	234	3	0.19

The genes identified as differentially expressed were analyzed by gene set enrichment analysis, using the same method also used elsewhere [24]. In this way we could monitor the pathways showing a global transcriptional perturbation upon silencing of PSCA (Figure 3B). We defined as up- or down-regulated the pathways statistically enriched in the list of genes up- or down-regulated, respectively, in the presence or absence of treatment. A statistically significant enrichment was observed between the list of genes up-regulated upon PSCA silencing (dox+). The IFNα/β signaling pathway resulted to be up-regulated upon PSCA silencing in the *in-vivo *experiments but its up-regulation was not evident in the *in vitro *datase. Out of the 27 genes listed in the Ingenuity database for interferon signaling 12 were up-regulated *in viv*o and 1 *in vitro*, STAT2. Similar results were obtained using the GeneGo database to run the analyses (8 out of 28 genes). In addition, the "antigen presentation" pathway resulted as perturbed with the highest statistical significance. Other immune related pathways were also affected although at lower statistical significance. In agreement with the reduced cell viability *in vitro *(Figure 1B) and reduced tumor growth (Figure 2A) the down-regulated pathways were "growth" and "negative regulation of apoptosis".

**Figure 4:**
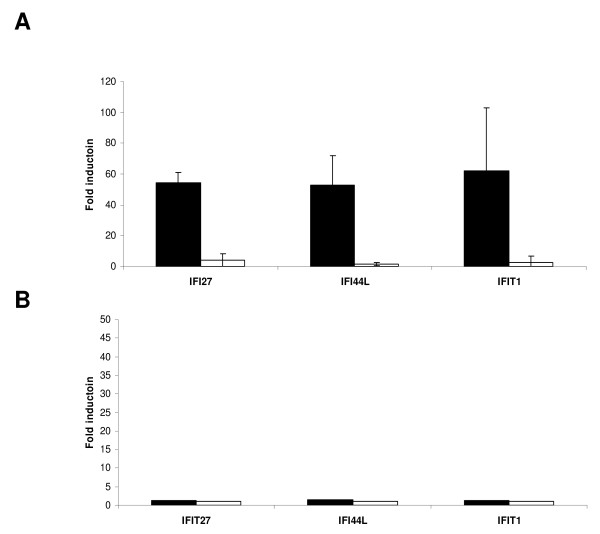
**Expression levels of selected IFNα/β pathway genes as a function of PSCA expression**. Expression levels for IFI27, IFI44L and IFIT1 genes were measured in individual SW780-shPSCA tumors (n = 6-8) **(A) **or in SW780-shPSCA in cell culture conditions **(B) **with or without dox treatment. Gene expression was measured by qPCR using the ΔΔ ct method. The average gene values in dox untreated mice served as reference and GAPDH gene as normalizer. In black average values in SW780-shPSCA tumors and in white SW780-shCont.

To measure by a more quantitative method the amplitude of gene regulation, selected genes were analyzed by qRT-PCR. As shown in Figure 4A, a subset of genes belonging to IFNα/β pathway such as IFIT1, IFI27 and IFI44L showed a statistical significant up-regulation (p < 0.001) in tumors where PSCA was down-regulated by dox treatment. To exclude that expression of shRNA per se could trigger expression of IFN pathway genes, tumors expressing control shRNA were analyzed. No statistical differences were observed with or without control shRNA expression. Interestingly, qPCR analysis of SW780-shPSCA in cell culture (Figure 4B) confirmed the lack of induction of the IFNα genes regardless of PSCA expression, suggesting a distinct role for PSCA in the context of tumor growth.

## Discussion

In this study, we have identified a correlation between PSCA expression and tumor growth *in vivo*. High level of PSCA mRNA and protein expression has been observed in most primary prostate and pancreatic human tumors and in particular in aggressive metastatic forms. In bladder cancer, higher levels of PSCA expression correlated with increasing tumor grade [25] and more recently genetic variants in this gene were associated with cancer occurrence, although the biological implications of this observation remain to be elucidated[26].

The observed tumor specific expression of PSCA has prompted the development of therapeutic antibodies specific for this membrane protein. Indeed, growth inhibition has been observed upon antibody treatment of cells transfected with vectors expressing PSCA or of naturally expressing cell lines such as SW780. The published studies are in agreement with the data reported here indicating that reduced PSCA expression is associated with lower viability [7]. Thus, based on these published data, a better understanding of the biologic role played by PSCA in tumor growth is warranted.

To shed light on PSCA role in tumor biology we compared PSCA expression in different cell lines and found expression on cell surface in few of them or in explanted primary tumors. Thus, the bladder cell line SW780 was engineered to obtain a pharmacological controlled expression of PSCA and to study the impact of reduced PSCA gene expression on cell viability and tumor growth. Here, we report a direct correlation between PSCA expression and tumor growth. Of note is the observation that pretreatment with dox *in vitro *further reduced tumor growth. This latter result may be partially explained by the slow decay of PSCA displayed on the cell membrane as indicated by FACS analysis.

A role for PSCA in tumor proliferation is further supported by the biological pathway analysis of gene expression profiling where a statistical significant association with "negative regulation of apoptosis" and "growth" was observed. Many of the pathways activated upon PSCA downregulation control key immune functions. Activation of IFNα/β signal transduction pathway was observed only in SW780-shPSCA tumors (Figure 2B) and it was not evident in tumors obtained upon injection of control cells. In contrast, similar studies conducted with these cell lines *in vitro *did not show induction of the pathway suggesting that in the tumor context additional factors such as those related to immune modulation may contribute to the observed microarray signature. A quantitative analysis of gene expression by qPCR confirmed overexpression of IFNα/β genes (Figure 4). The activation of IFNα/β pathway only in vivo further supports the idea that triggering of this pathway is not determined by an intracellular mechanism related to double strand RNA, as it has been previously reported. On the contrary, the data suggest that IFNα/β activation is related to environmental signals. The activation of IFNα/β pathway is consistent with a reduced tumor growth as previously shown with recombinant IFNα [27,28] .

Nonetheless, further experiments are required to better characterize the link between PSCA downregulation and activation of immune pathways such as IFNα/β. In this direction it is worth mentioning a potential physical interaction between PSCA and IFNα/β receptor. PSCA is a GPI-anchored protein located in lipid raft. IFNα/β receptor can be brought into this subcellular compartment upon interaction with its ligands [29]. Thus, PSCA may counteract intracellular signaling exerted by the IFNα/β receptor playing a role as a defense protein. This biological role is reminiscent of the interfering role played by PSCA homolog, CD59, in the complement system [30]. Activation of the complement system is tightly regulated and among the various regulators CD59 has been identified as the single membrane regulator of the terminal membrane attack complex. Moreover CD59 has been characterized as a negative regulator of the T-cell response in mice. Although we limited our qPCR confirmation only to IFNα/β pathway, a statistical significant up regulation was observed also for other immune pathways such as of MHC-1 pathway. Given the pleiotropic effects of IFNα/β pathway it is likely that some pathways, including MHC-1 presentation, may be affected downstream. A more in depth analysis would be required to answer this question. Finally we cannot exclude that the correlations observed only in tumors and not in cell culture are driven by undefined factors that are not captured in the expression profile and deserve further investigation.

## Conclusions

The current findings suggest a role of PSCA in counteracting the natural immune response against cancer. This information could help in design more effective experiments in preclinical models not only with monoclonal antibodies but also with approaches based on cancer vaccine [31-35]. It would be of interest to correlate antitumor efficacy of PSCA-targeting therapies as a function of the natural immune response.

## Competing interests

The authors declare that they have no competing interests.

## Authors' contributions

EM generated cell lines expressing shRNA, analyzed PSCA expression by FACS analysis and performed experiments in vivo. PU performed microarray analysis and pathway analysis- VV generated cell lines expressing shRNA and performed IHC on tumor samples. VS performed qPCR analysis. ED contributed to the discussion of the data. EDR contributed in the discussion of the bioinformatics data and helped in drafting bioinformatics result section. AL performed the initial microarray analysis and contributed to data analysis. NLM, AN and GC contributed to the drafting and general discussion of the paper. FP conceived the study, analyzed the data and wrote the manuscript. All authors read and approved the final manuscript.

## Pre-publication history

The pre-publication history for this paper can be accessed here:

http://www.biomedcentral.com/1471-2407/10/129/prepub
